# Dynamic Threshold Selection for a Biocybernetic Loop in an Adaptive Video Game Context

**DOI:** 10.3389/fnhum.2018.00282

**Published:** 2018-07-17

**Authors:** Elise Labonte-Lemoyne, François Courtemanche, Victoire Louis, Marc Fredette, Sylvain Sénécal, Pierre-Majorique Léger

**Affiliations:** Tech3Lab, HEC Montréal, Université de Montréal, Montreal, QC, Canada

**Keywords:** dynamic adaptation, passive brain computer interface, hybrid brain computer interface, BCI, pBCI, hBCI, EEG, tetris

## Abstract

Passive Brain-Computer interfaces (pBCIs) are a human-computer communication tool where the computer can detect from neurophysiological signals the current mental or emotional state of the user. The system can then adjust itself to guide the user toward a desired state. One challenge facing developers of pBCIs is that the system's parameters are generally set at the onset of the interaction and remain stable throughout, not adapting to potential changes over time such as fatigue. The goal of this paper is to investigate the improvement of pBCIs with settings adjusted according to the information provided by a second neurophysiological signal. With the use of a second signal, making the system a hybrid pBCI, those parameters can be continuously adjusted with dynamic thresholding to respond to variations such as fatigue or learning. In this experiment, we hypothesize that the adaptive system with dynamic thresholding will improve perceived game experience and objective game performance compared to two other conditions: an adaptive system with single primary signal biocybernetic loop and a control non-adaptive game. A within-subject experiment was conducted with 16 participants using three versions of the game Tetris. Each participant plays 15 min of Tetris under three experimental conditions. The control condition is the traditional game of Tetris with a progressive increase in speed. The second condition is a cognitive load only biocybernetic loop with the parameters presented in Ewing et al. (2016). The third condition is our proposed biocybernetic loop using dynamic threshold selection. Electroencephalography was used as the primary signal and automatic facial expression analysis as the secondary signal. Our results show that, contrary to our expectations, the adaptive systems did not improve the participants' experience as participants had more negative affect from the BCI conditions than in the control condition. We endeavored to develop a system that improved upon the authentic version of the Tetris game, however, our proposed adaptive system neither improved players' perceived experience, nor their objective performance. Nevertheless, this experience can inform developers of hybrid passive BCIs on a novel way to employ various neurophysiological features simultaneously.

## Introduction

We have become quite adept at communicating with our computer systems and having them do what we wish. However, this type of communication lacks the subtleties that accompany human-human interactions through non-verbal signals. A promising new mode of communicating with technology has emerged that could help our computer systems understand more understated information about us, and that is passive brain-computer interfaces (pBCIs) (Zander and Kothe, 2011). This paper presents suggested improvements to the current state of pBCI development.

With pBCIs, the user's state is communicated to the computer without any conscious effort from the user and the system takes that into account to adapt itself to ultimately lead the user to an optimal state. Passive BCIs can bring about many different types of adaptations, such as modifying the level of difficulty of a game to ensure it is as stimulating as possible for players (Van De Laar et al., 2013), set off an alarm when truck drivers are too tired to be safe on the road (Hajinoroozi et al., 2016), or disengaging the autopilot to prevent airline pilots from getting so bored that they fail to notice important warning signs (Pope et al., 1995). Passive BCIs, as opposed to active BCIs, are mostly being developed for average users to improve their daily experiences rather than to replace other input devices such as using eye tracking instead of a mouse (Frisoli et al., 2012). As mentioned by Robert Jacob, “active BCIs have the potential to bring a great improvement to the lives of a small number of people, while passive BCIs can bring small improvements to the lives of a great number of people” (Jacob, 2017, p. 14).

While multiple research groups have been building functional pBCI systems (Prinzel et al., 2000; Scerbo et al., 2003; Berka et al., 2004; Lin et al., 2006; Ewing et al., 2016), many technical challenges remain to be addressed (Allanson and Fairclough, 2004; Fairclough, 2011; Brouwer et al., 2015), such as personalization. As biosignals vary greatly from one person to another, personalization is required for systems to function properly (Makeig et al., 2012). Developers will often use personalization tasks to individually tailor thresholds before the use of a system (Johnson et al., 2011). Their participants will thus have to be recorded twice, once to measure their responses to a task designed to induce a given state and a second time once the system has been personalized to actually use it. This requires time and resources and results in highly personalized systems that are, nonetheless, not responsive to changes over time, such as fatigue. This is the challenge we attempt to address within this paper.

A solution that has been proposed to many such BCI problems is the combination of multiple neurophysiological signals (hybrid BCI) to improve reliability, proficiency, bandwidth, convenience, and utility (see Banville and Falk, 2016 for a review; Nijholt et al., 2011). The type of system presented in this article can incorporate both neurophysiological and psychophysiological measures into the same system (Pfurtscheller et al., 2010; Zander et al., 2010; Banville and Falk, 2016). It is also commonly known as multimodal BCI. We look to the hybrid BCI to personalize our system in real-time and diminish the time and resources that are required before a user can begin using a system for the first time. While most hybrid BCIs use a second signal to improve the robustness of the system, to our knowledge, no other group has used a secondary neurophysiological signal to adjust the parameters of the system's adaptation to the first signal. Basically, the second signal would be used as a successfulness indicator of the adaptation, which is based on the first signal, leading it to be personalized. To our knowledge, this paper presents a first attempt to do so.

Our approach takes a slightly different view of pBCIs as most systems intend the subject to be unaware of the adaptations taking place. We propose instead that subjects do not need to be completely conscious of the changes, but that they are probably, on some level, aware that changes are taking place. By measuring the impact of these changes with a secondary, affective signal, we intend to measure the user's reaction to the adaptation in real time. This should inform the system whether the adaptation improved their experience (and/or performance) or not, thus telling it whether the adaptation parameters were correct or not. Accordingly, we propose a new approach for dynamic threshold selection that performs a continuous calibration. The biocybernetic loop adapts to one physiologically inferred metric, in this case, cognitive load, using electroencephalography (EEG), while the thresholds are continuously adjusted based on a second metric, in this case, emotional valence, inferred using automatic facial expression analysis.

The goal of this paper is to evaluate if dynamic thresholding with a secondary neurophysiological signal can continuously personalize the adaptive system. We hypothesize that the adaptive system with dynamic thresholding will improve perceived game experience and objective performance.

## Methods

### Participants

This study was carried out in accordance with the Research Ethics Board of our institution and with written informed consent from all subjects. Participants were students at local universities. There were 16 in total (6 women). Average age was 27.75 (*SD* = 8.55). Participants had to be over 18 and have no known health issues. They received a $30 gift card as a compensation.

### Initial baselining

We used a modified vanilla baseline task (Jennings et al., 1992) as a baseline calibration task. This was chosen because it is an emotionally neutral task with similar visual characteristics as the Tetris game, i.e., color and movement.

### Experimental design

As Ewing et al. (2016) underlined, game periods need to be longer than 5 min to get an accurate representation of the system's adaptation. Thus, participants played each condition for 15 min. The order of the three conditions was counterbalanced between participants. For the adaptive conditions, the system managed the game difficulty as in the Dynamic Difficulty Adjustment framework (Hunicke and Chapman, 2004).

### Game

Tetris is a game that is often used in research as it is easily adaptable and because gameplay is generally understood by most. In this instance, Tetris was also advantageous as there is no emotional component to the gameplay. This prevented us from having to tease out the effect of gameplay from the effects of the adaptive system on the measured valence, the latter being more relevant to our study.

To easily control game parameters, we developed an in-house version of Tetris. The game window consisted of a rectangle of 10 by 20 squares. Players controlled the game with a Logitech F310 gamepad (Logitech, Lausanne). They could move the pieces laterally with the left and right arrows and rotate the pieces with the “A” and “X” buttons. No other manipulation was allowed.

For each condition, the initial speed was 400 ms per line. The slowest allowed speed was 1,500 ms per line and the fastest allowed speed was 100 ms per line. Speed variations between levels were of 100 ms at a time. These parameters were chosen after pilot testing was conducted. The only parameter that was atypical was the absence of the control that allows the user to manually make the blocks fall faster. The ability to force the blocks down which might prevent the conditions from being uniform between subjects, thus it was not implemented in our game.

#### Condition 1

In the control condition, there was no adaptation taking place. The speed increased by one step (speed levels are defined in the next section) every time four rows were successfully cleared. This condition was chosen to resemble a traditional game of Tetris as closely as possible.

#### Condition 2

**Figure 1:**
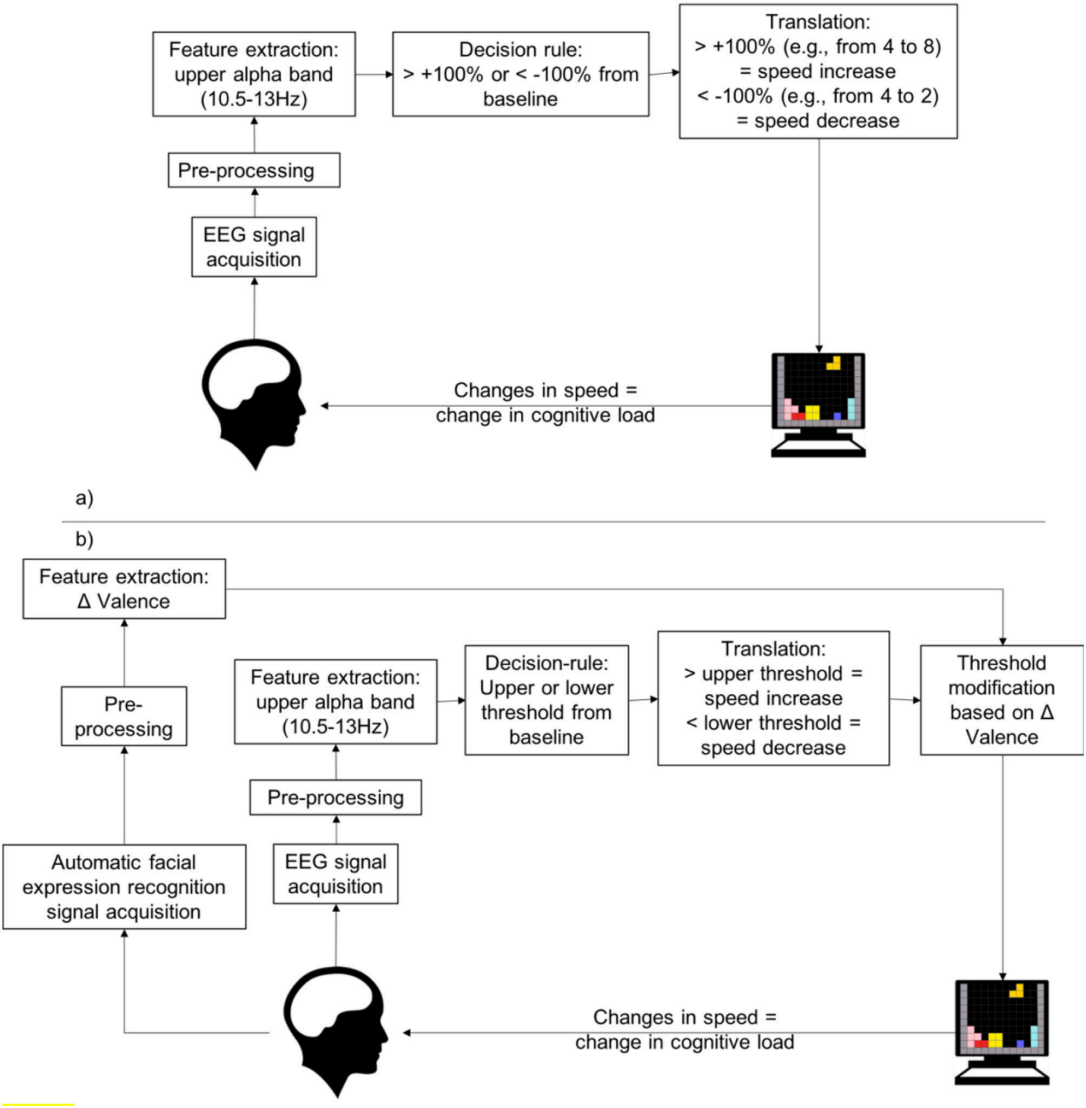
**(a)** Flowchart of the pBCI in Condition 2. The game speed adapts according to changes in the EEG from baseline. In this condition the threshold at which changes in the EEG register as sufficient to trigger a change in speed is fixed at 100%. **(b)** Flowchart of the pBCI with adaptive thresholds in Condition 3. The game speed adapts according to changes in the EEG from baseline. In this condition, the threshold at which changes in the EEG register as sufficient to trigger a change in speed is continually adjusted according to the valence changes surrounding the previous speed changes.

The second condition was developed to model as closely as possible to Ewing et al. (2016) conservative condition. The system adapts to the user state as measured by the EEG. Any 100% or two-fold increase/decrease from the baseline value (e.g., for a starting baseline value of 4, a 100% increase would be a value of 8, a 100% decrease would be a value of 2) or more from the benchmarking task triggers a speed change (see Figure 1a). Ewing et al. (2016) employed the upper alpha (10.5–13 Hz) in the P4 location and theta bands (4–8 Hz) in the Fz location in the following manner: an increase of theta accompanied by an increase in alpha (both should be more than 100% baseline) corresponds to the user being in a state of boredom and the speed is increased; an increase of theta accompanied by a decrease in alpha (both 100% or more) corresponds to the user being in a state of overload and the speed is decreased. Similarly, our system adaptations of speed are triggered by 100% or more changes to the baseline level of the upper alpha band (10.5–13 Hz). This band was chosen from Ewing et al. (2016) following pilot testing.

#### Condition 3

**Figure 2:**
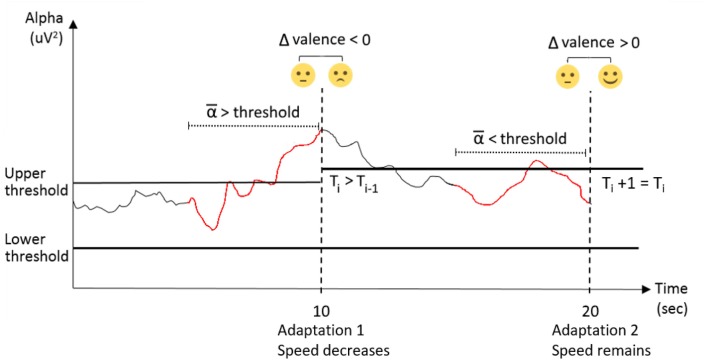
Illustration of the dynamic thresholds adaptation in four steps (adaptation pacing = 10 s): (1) The average of alpha over the 5 s (in red) preceding adaptation 1 is higher than the upper threshold; (2) The system therefore choose to decrease the game speed at adaptation 1; (3) The difference in valence before (average of 2 s) and after (average of 2 s) adaptation 1 is negative; (4) The system qualifies adaptation 1 as a fail and raises the upper threshold in order to be less sensitive during adaptation 2. T_i_ refers to the threshold value at the offset of a given adaptation (i).

The third condition is similar to the second condition (see Figures 1a,b for a side by side comparison) with the addition of adaptive thresholds. The thresholds adapt to the emotional valence of the subject, i.e., the extent to which an emotion is positive (Lang et al., 1995). Valence is measured with the use of facial expression analysis, which is explained in the next section. The emotional valence is measured before and after each speed adaptation over 2 s. If the after-before delta is positive, the subject is deemed satisfied with the adaptation, the threshold value is considered appropriate and is maintained. If the delta is negative, the subject is deemed dissatisfied with the adaptation and the threshold value is modified as follows. There are two threshold values, the lower threshold which, when crossed, leads to an increase in speed, and the upper threshold, which, when crossed leads to a decrease in speed. When the subject is deemed dissatisfied with the adaptation, only the threshold that was crossed is changed. The thresholds move away from the middle, i.e., the upper threshold increases, or the lower threshold decreases, leading to a less severe adaptation criteria (see Figure 2). For example, if the upper threshold value was 1, and the baseline alpha value was 4, when the current alpha value reaches past 8 for a given window (a 100% increase), it has crossed the threshold. Thus, the decision-rule determines that the game speed must slow down. At the moment where the speed is modified, the delta valence is measured. The valence value from before the speed change is subtracted from the valence value after the speed change to see if the change is received positively or negatively. For example, if the valence before the speed change was 2 and the valence after the speed change is 1, the delta valence is negative (−1). The threshold value from our previous example, a value of 1 for the upper threshold, should be modified. It will move away from the center; therefore, the upper threshold value will increase to 1.1. In the next window, for the speed to change, the alpha value will need to reach 8.4 (an increase of 110% of the baseline value of 4). In addition, when, for a given 10 second epoch, no adaptation takes place while delta valence is negative, both thresholds are considered too loose and are brought closer to the middle, i.e., the upper threshold decreases, and the lower threshold increases, leading to a more severe adaptation criterion.

### Measurement tools and processing

#### EEG recording and processing

The EEG was recorded from 32 Ag-AgCl preamplified electrodes mounted on the actiCap and with a brainAmp amplifier (Brainvision, Morrisville). The recording reference was FCz and the acquisition rate was 500 Hz. All the EEG processing was performed in the NeuroRT software (Mensia, Renne). The following steps were performed in this order for the online preprocessing of the data: down-sampling to 256 Hz, bandpass filtering with an infinite impulse response filter at 1–50 Hz, notch filtering at 60 Hz, blink removal through blind source separation, re-referencing to the common average reference, and artifact detection by computing the Riemannian distance between the covariance matrix and the online mean. The data was then filtered to keep only the upper alpha band (10.5–13 Hz) and squared. Only data from P3 and P4 was kept in accordance with Ewing (Ewing et al., 2016). Finally, each 0.5 s epoch was divided by the average of the 1-min calibration period to normalize the data. This value, which ranged from 0.008 to 6.87, was then sent to the adaptation system to be used in the decision rule-based process.

#### Facial expression analysis

Emotional valence was obtained through the use of facial expression analysis performed in real time with the FaceReader software (Noldus, Wageningen). Automatic facial expression analysis uses the Facial Action Coding System (Ekman et al., 1980) that has traditionally been employed by human experts who manually code video playback for facial expressions. Specific combinations of muscle contractions are associated to certain emotions. Computer image recognition is now able to detect these same muscle contraction combinations and code the user's emotions in real time (Bartlett et al., 1999). We used the Facereader valence ratio which is calculated as the intensity of positive emotion minus the intensity of the negative expression with the highest intensity. The software maps the facial muscles of the user and derives valence at a rate of 30 Hz. It streamed to the adaptive system that used the 2 s before and the 2 s after an adaptation to calculate the change in valence caused by the adaptation. A positive change tells the system that the adaptation was appropriate, meaning that the threshold parameters were correct, while a negative change tells the system that the adaptation was wrong and that the thresholds should be modified.

### Game performance

The behavior of each system (Conditions 1–3) was logged in order to compare them and understand what drove user experience. The game speed was controlled as the duration (ms) it took for a block to move down one line. This was converted *post-hoc* into levels. The smaller the level, the slower the game and there was 100 ms difference between levels (i.e., level 1 = 1,500 ms, level 15 = 100 ms). The number of “game deaths” per subject and the score of the subject at the end of each condition were also logged (Table 1). Participants scored 100 points each time they cleared a line, 300 points for two lines, 500 points for three lines, and 800 points for four lines.

**Table 1:** High and low speed comparisons for each experimental condition.

	**Condition 1**	**Condition 2**	**Condition 3**
Alpha	NA	Low speed = 0.65 High speed = 0.82 *p* = 0.21	Low speed = 0.69 High speed = 0.86 *p* = 0.03
Valence	NA	Low speed = 0.03 High speed = 0.14 *p* = 0.21	Low speed = 0.03 High speed = 0.13 *p* = 0.11
Average game level	Low speed = 12.37 High speed = 12.69 *p* = 0.39	Low speed = 3.94 High speed = 7.69 *p* = 0.05	Low speed = 4.41 High speed = 10.84 *p* < 0.001
Game deaths	Low speed = 3.6 High speed = 3.9 *p* = 0.84	Low speed = 0.3 High speed = 1.3 *p* = 0.72	Low speed = 0.14 High speed = 1.3 *p* = 0.09
Score	Low speed = 4,186 High speed = 5,367 *p* = 0.35	Low speed = 2,043 High speed = 2,989 *p* = 0.26	low speed = 1957 high speed = 956 *p* = 0.008

### Perceived measures

In addition to game performance, we also measured the perception of the participant at the end of each experimental condition. We used the In-game and Post-game Game Experience Questionnaire (GEQ), a shortened version of the core questionnaire which was developed to assess multiple iterations of an experiment (IJsselsteijn et al., 2008). These questionnaires respectively have 14 and 17 items that lead to scores on the following dimensions: competence, flow, annoyance, challenge, positive, and negative affect are the 6 dimensions of the In-game GEQ and positive experience, negative experience, tiredness, and returning to reality are the 3 dimensions of the pots-game questionnaire. Each item has a 5-point scale ranging from 0 (Don't get the feeling described in the statement at all) to 4 (Extremely get the feeling described in the statement).

### Statistical analysis

Diverse statistical tests have been performed between the three conditions (control, EEG, adaptive thresholds) and between clusters of participants afterwards. In order to compare conditions (GEQ, total scores, average game level, valence, alpha power), we used Wilcoxon tests to compare the 3 conditions and Mann–Whitney tests to compare the clusters of participants. Both these tests are non-parametric as is appropriate considering the sample size. Also, a Kolmogorov–Smirnov test was used to test for normality and found only one variable to be normally distributed, the alpha power in conditions B and C (D = 0,12, *p* = 0.15; D = 0.16, *p* = 0.15) further justifying the use of non-parametric tests. We used Poisson regressions with random effects to account for repeated measures from the same participant to compare total number of deaths and total number of level changes between the 3 conditions.

## Results

**Figure 3:**
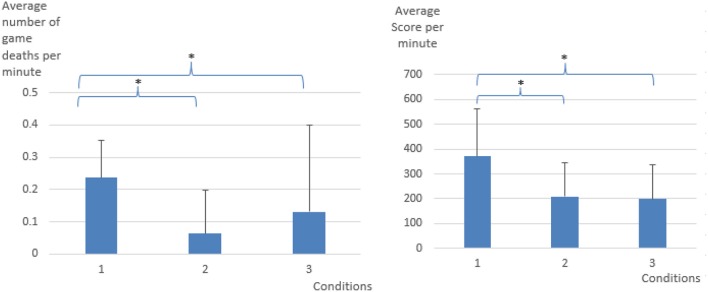
Average number of game deaths per minute and average score per minute per experimental condition. Error bars represent standard deviation. *p < 0.05.

**Figure 4:**
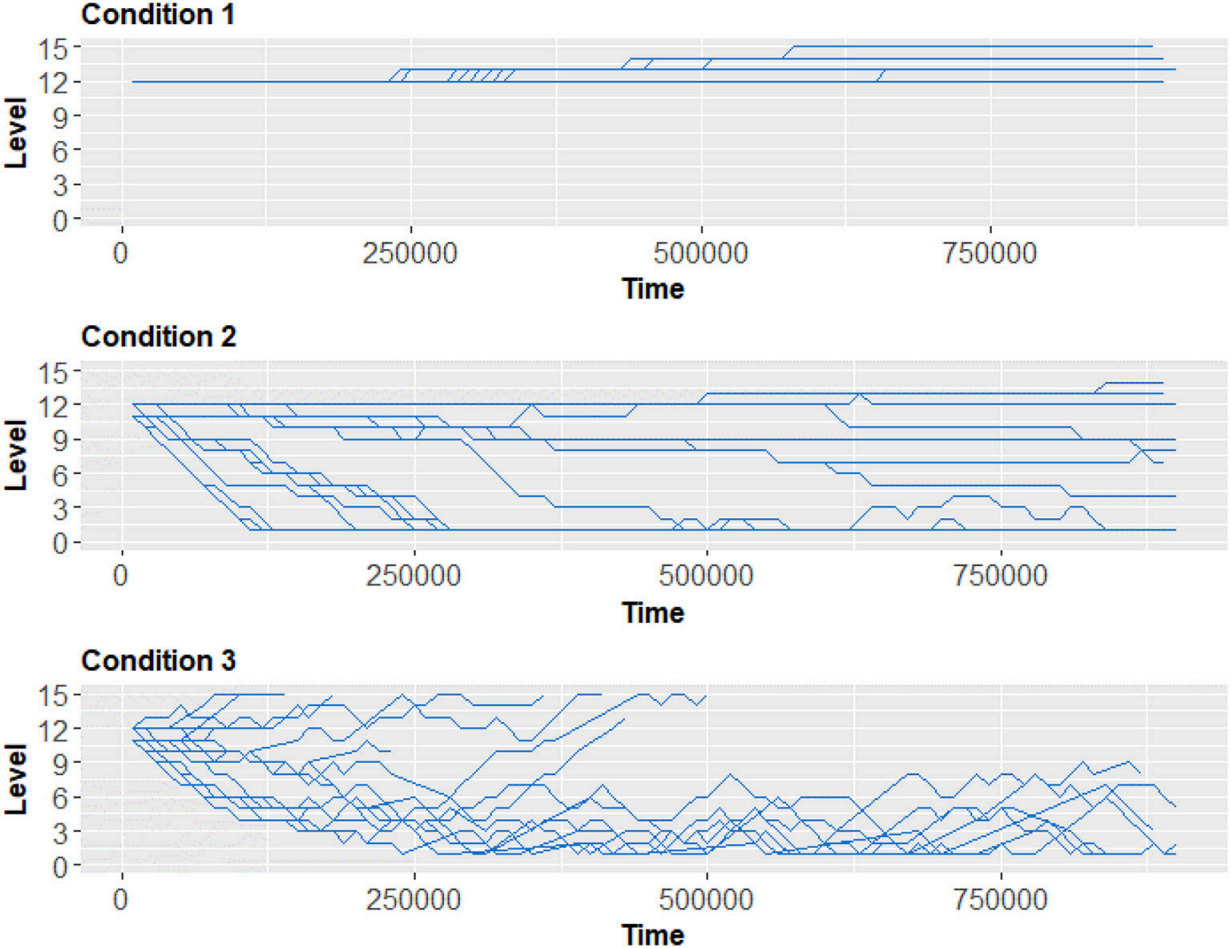
Average game level for each experimental condition. Each line is the game level of a study participant over time in milliseconds. A higher game level means that the speed at which the blocs descend increases, thus diminishing the time available to position the bloc properly and increasing difficulty. In Condition 1, participants began at level 12 and the level increased progressively. In Condition 2, for most participants the level decreased over time. In Condition 3, a split appears, where some participants see their level increase irreversibly, while other participants have a result more similar to Condition 2. In a functioning system, there would be a lot of variations early on and after some time the levels would stabilize and adjust linearly. For Condition 3, we present only results for which the adaptive mechanism leads to thresholds that were mathematically attainable. This issue is discussed in further details in the next section.

As can be seen in Figure 3, when compared to the control condition (Condition 1), the number of game deaths was significantly lesser in the EEG only BCI (Condition 2), and in the adaptive thresholds condition (Condition 3). The score, when compared with the control condition, was lesser in both BCI conditions. The average game level (seen in Figure 4) in each condition was 12.53 for control (Condition 1), 6.18 for EEG only (Condition 2), and 8.92 for adaptive thresholds (Condition 3). All differences were significant at *p* < 0.05. These results show that both BCI conditions seem to have “over adapted” the game resulting on average in slower games (lower game level) and decreased player performance (score) compared to the control condition.

**Figure 5:**
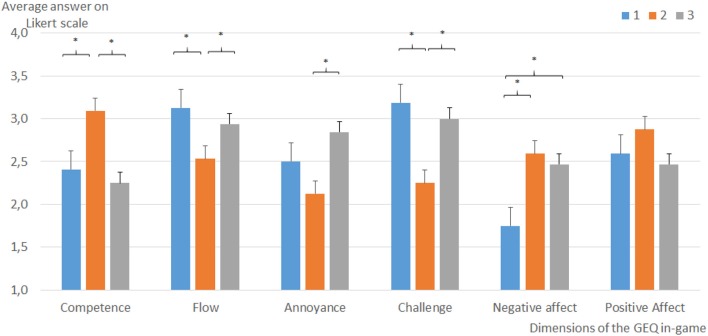
Average answers to the in-game portion of the Game Experience Questionnaire (GEQ) showing the results for each dimension measured. Error bars represent standard deviation. *p < 0.05.

**Figure 6:**
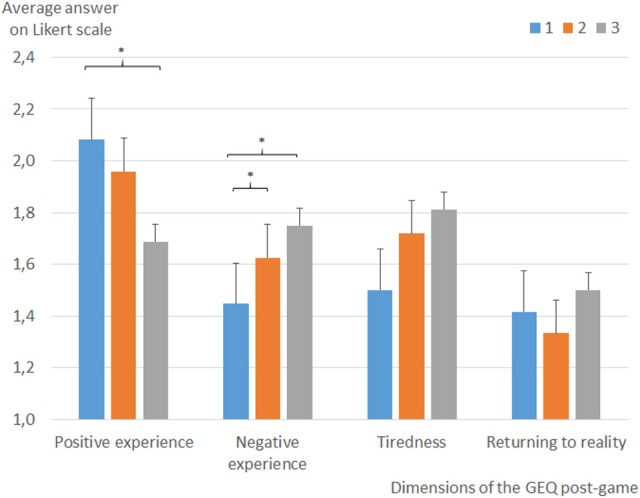
Average answers for the post-game portion of the Game Experience Questionnaire (GEQ) results showing the results for each dimension measured. Error bars represent standard deviation. *p < 0.05.

As for the GEQ, results and significant differences can be seen in Figures 5, 6. Participants in the EEG condition (Condition 2) perceived the experience as less challenging (comparison 1 vs. 2, *p* = 0.004; comparison 2 vs. 3, *p* = 0.059), negatively charged (comparison 1 vs. 2 *p* = 0.012), and less conducive to flow (comparison 1 vs. 2, *p* = 0.019; comparison 2 vs. 3, *p* = 0.086) compared to the two other conditions. The fixed threshold condition (Condition 3) is perceived as less annoying (comparison 2 vs. 3 *p* = 0.032) and participants in this condition develop a higher perceived competence than in the two other conditions (comparison 1 vs. 2 *p* = 0.008; comparison 2 vs. 3 *p* = 0.033). Participants felt the experience in Condition 1 more negatively than in the two other conditions (comparison 1 vs. 2 *p* = 0.031; comparison 1 vs. 3 *p* = 0.041).

When explored further, our results appeared to show two different clusters of responses in Condition 3. Thus, we evaluated how many subjects spent 5 or more periods of 10 s at the fastest speed or at the slowest speed. Once these two groups were identified, we compared them with Wilcoxon tests to evaluate if they differed in their alpha power change from benchmark, valence, GEQ, number of game deaths, and game scores.

Results suggest that in Condition 3, 9 participants spent a large portion of their time at the slowest speed, while 10 participants spent most of their time at the fastest speed. Only three participants spent <25 s at either extremity. When compared, these two groups (slow speed group vs. high speed group, Condition 3) are statistically different for their average EEG alpha power change from baseline (*p* = 0.01), where the high-speed group has more alpha (high speed = 0.99; low speed = 0.67). They are also statistically different in terms of valence values (*p* = 0.02; high speed = 0.01; low speed = 0.20).

We also evaluated if participants differed in Condition 2 to see if they were intrinsically different or if they were only different while using the dynamic threshold adaptive system. For those two groups (high and low-speed), their values did not differ in Condition 2 (alpha *p* = 0.35; valence *p* = 0.23; Table 1). Also, these groups differed on some dimensions of the GEQ. In Condition 2, they reported different annoyance perceptions (low speed = 2.78; high speed = 1.7; *p* = 0.02) and in Condition 3, challenge (low speed = 2.17; high speed = 3.65; *p* = 0.03) and competence perceptions differed (low speed = 3.22; high speed = 1.35; *p* < 0.01). Finally, the two groups significantly differed in game deaths in Condition 3 (high speed = 0; low speed = 19) and score in Conditions 1 and 2 (respectively, low speed = 4028.57, high speed = 5233.33; *p* = 0.001 and low speed = 2028.57, high speed = 2,700; *p* = 0.001).

## Discussion

Our results show that the adaptive systems did not improve the participants' experience while playing Tetris. Contrary to our expectation, participants had more negative affect from the fixed threshold and the dynamic thresholds conditions than in the traditional Tetris game (control condition). In the EEG only condition, similarly to Ewing et al. (2016), participants had fewer game deaths, a lower score, and a lower average speed; showing that the system slowed down the game, leading to the participants feeling more confident, but less challenged and ultimately feeling more negative affect.

If we examine the adaptive threshold condition by separating the participants according to how much time they spent at each extreme level, we can see that participants have significantly different alpha and valence. However, they did not differ in EEG only condition, showing that they are not fundamentally different in their neurophysiology, but that their neurophysiological responses to the use of an adaptive system were different. Obviously, the high-speed group found the challenge higher and their competence lower as they were generally unable to keep up with the game and died many more times than the low speed group.

One potential explanation of our counterintuitive results could be found in Csikszentmihalyi's flow theory (Nakamura, 2014). This specific flow theory postulates that an optimal positive valence experience emerges in an autotelic context when one's competence is in alignment with a given challenge. In self-motivating contexts, such as games, the theory predicts that when the challenge is above one's competency, a state of anxiety and frustration is likely to emerge. As the competency of the player evolves in addressing this challenge, the theory suggests that the individual is likely to move back toward a positively valenced equilibrium state. In our study, it is possible that the parameters to capture the change in the valence might not have been optimized to account for the time it takes for the player to come back to this equilibrium. This would suggest that future research needs to not only personalize the values that trigger adaptation, but also needs to personalize the adaption window duration. This could be done through a more complex baseline task that would be able to capture this idiosyncratic latency in players.

With both these personalizations in place (threshold and adaptation time), we could hope to reach both subject independence and context independence. Subject independence and context independence are the big challenges currently facing passive BCIs in their quest for use in everyday life (BNCI Horizon 2020, 2015). The hope is to have a system that can be simply put on the user's head and work straight away in a variety of contexts without the need for lengthy calibration tasks. Our goal was to develop a system that continuously adjusts its action triggering threshold rules which would have been a step in the direction of subject independence, or at the least, a system that is subject dependent and context independent. While our system needs more testing and calibration to evaluate its feasibility, we trust that continuous adaptation and the use of multimodal neurophysiological signals hold the key to those challenges. Other researchers should keep in mind that the use of valence-based tools may differ in the context of games with more emotionally charged objects and not extrapolate our results to those contexts. Future works will explore more comprehensive EEG data than alpha alone, and various window durations to see if the affective response to the speed adaptation appears after a delay, or if it may simply take some time for the user to get comfortable at a given level thus the speed changes should happen less frequently. In addition, as speed changes in quick succession are not expected when playing Tetris, perhaps novelty played a role in the user's dissatisfaction, thus a variety of adaptation windows could be tested.

While evaluating a variety of neurophysiological data sources and time windows could lead to a solution, other possible avenues of exploration have emerged. As mentioned in the literature, machine learning would greatly improve this type of system (Scherer et al., 2013; Brouwer et al., 2015; Ewing et al., 2016). On one side, recent advances in deep learning techniques could help enhance the accuracy of cognitive state inference models. Deep learning models are well suited to find complex hierarchical patterns in high dimensionality data such as neurophysiological data. Research using such approaches for psychophysiological inference (Martinez et al., 2013) or EEG signal decoding (Schirrmeister et al., 2017) show promising results. On the side of the interaction loop, we would recommend the use of reinforcement learning in the case of hybrid BCIs. Reinforcement learning uses a problem space to determine where it is in relation to the optimal location. The system adapts within this problem space and gets rewarded as it gets closer to the goal. In the case of our proposed biocybernetic loop, the problem space would be defined as a grid of difficulty by neurophysiological state and the reward could be determined by a second neurophysiological signal. Consider the system presented in this paper. As a reinforcement learning system, the problem space in which the model would evolve and try to find an optimal path would be composed of the two dimensions of cognitive load and current speed of the game. Each action undertaken by the model would be rewarded by the local changes in the subject's valence following the adaptation. Doing so, the system should converge toward an optimal state of difficulty vs. cognitive load that is specific to each subject and that may change over time. In this approach, the adaptation thresholds would be replaced by the transitioning policy optimized over time that the system uses to decide which action to perform. We can imagine a similar system with a problem space composed of simulator challenge and vigilance level with a reward based on stress levels. The idea behind this is that an affective index could confirm or infirm if the action performed by the system improved the user experience in real time instead of having to validate with the user verbally after they test it for a while and adjusting the system manually afterwards. Our future work will explore this promising alternative.

We endeavored to develop a system that improved upon the authentic version of the Tetris game, however, our proposed adaptive system neither improved players' perceived experience, nor their objective performance. In practice, for condition 3, the way we modified the threshold values based on valence could eventually lead to values that were impossible to attain. For some participants, the thresholds crossed, leading to some lower thresholds being higher than upper thresholds and for some thresholds to be negative which is impossible to attain from the alpha ratio. Nevertheless, this experience can inform developers of hybrid passive BCIs on a novel way to merge various neurophysiological features. If the goal of a passive BCI system is to be widely adopted, it must be better than the current version of a similarly oriented system. Comparisons to other versions of a given BCI (Liu et al., 2009; Chanel et al., 2012) are very informative to better understand how parameters of the BCI influence the user-system relationship, however, this is only one part of the picture when it comes to the future adoption of passive BCIs in everyday lives. As mentioned by Nijholt et al. (2011), new types of human-computer interaction take years of research before they come into their own, thus all advances should be presented and discussed. Ultimately, the true test will be to compare any new system to the status quo in order to improve it.

## Author contributions

EL-L and FC designed the study and developed the system. All authors contributed to the analysis and the writing.

### Conflict of interest statement

The authors declare that the research was conducted in the absence of any commercial or financial relationships that could be construed as a potential conflict of interest.
